# Development of an LC-HRMS/MS Method for Quantifying Steroids and Thyroid Hormones in Capillary Blood: A Potential Tool for Assessing Relative Energy Deficiency in Sport (RED-S)

**DOI:** 10.3390/metabo14060328

**Published:** 2024-06-12

**Authors:** Chiara Tuma, Andreas Thomas, Hans Braun, Mario Thevis

**Affiliations:** 1 Institute of Biochemistry, Center of Preventive Doping Research, German Sport University Cologne, 50933 Cologne, Germany; 2 German Research Centre of Elite Sports (Momentum), German Sport University Cologne, 50933 Cologne, Germany; 3 European Monitoring Center for Emerging Doping Agents (EuMoCEDA), 50933 Cologne, Germany

**Keywords:** microsampling, VAMS, mass spectrometry, energy availability

## Abstract

Relative energy deficiency in sport (RED-S) is a condition that arises from persistent low energy availability (LEA), which affects the hypothalamic–pituitary axis and results in alterations of several hormones in both male and female athletes. As frequent blood hormone status determinations using venipuncture are rare in sports practice, microsampling offers promising possibilities for preventing and assessing RED-S. Therefore, this study aimed at developing a liquid chromatography–high-resolution tandem mass spectrometry (LC-HRMS/MS) method for quantifying relevant steroids and thyroid hormones in 30 μL of capillary blood obtained using Mitra^®^ devices with volumetric absorptive microsampling technology (VAMS^®^). The results of the study showed that all validation criteria were met, including a storage stability of more than 28 days in a frozen state (−18 °C) and 14 days at room temperature (20 °C). The validated assay provided precise (<12%) and accurate (<13%) results for all the target analytes. Furthermore, as a proof of concept, autonomously collected VAMS^®^ samples from 50 female and male, healthy, active adults were analyzed. The sensitivity of all analytes was adequate to quantify the decreased hormone concentrations in the RED-S state, as all authentic samples could be measured accordingly. These findings suggest that self-collected VAMS^®^ samples offer a practical opportunity for regular hormone measurements in athletes and can be used for early RED-S assessment and progress monitoring during RED-S recovery.

## 1. Introduction

Analysis of endogenous hormone levels in circulation is not only important for the prevention and diagnosis of several diseases or in sports drug testing but is also relevant in sports science and sports nutrition.

For instance, in subjects with relative energy deficiency syndrome (RED-S), regular assessment of different hormones can be beneficial because RED-S can lead to metabolic changes, including hormonal imbalances [1,2]. The main reason for this is chronic low energy availability (LEA), which influences the hypothalamic–pituitary axis, leading to alterations in several blood hormone concentrations in both female and male athletes. These affect sex hormones via the hypothalamus–pituitary–gonadal axis and thyroid hormones via the hypothalamus–pituitary–thyroid axis, as well as glucocorticoids via the hypothalamic–pituitary–adrenal axis [1,3].

In addition to alterations in hormone status, the long-term consequences of chronic LEA include reduced bone density, menstrual cycle irregularities, and reduced athletic performance [1,2,4].

However, there is no established method for early detection or prevention of RED-S because of the often nonspecific and nonmeasurable symptoms associated with it. In addition, when symptoms are more severe, such as amenorrhea or bone injuries, preventive measures may no longer be effective. Therefore, objective parameters, such as hormone measurements, offer great potential for the early detection of endocrine alterations and monitoring the progress of recovery processes from RED-S.

Hormone measurements are not typically conducted on a regular basis in sports practice. There are several reasons for this, including the requirement for trained medical professionals to perform venipuncture as well as the high costs and logistical challenges involved. A more practical approach is to utilize microsampling techniques, which eliminate the need for large-scale blood collection and the involvement of medical personnel. These techniques rely on capillary blood drawn from e.g., the fingertip for analysis and can be used by athletes autonomously or by trained professionals, such as sports scientists or nutritionists.

Mitra^®^ devices with volumetric absorptive microsampling technology (VAMS^®^) are novel devices consisting of an absorbent polymeric tip mounted on a plastic holder unit. This device is specifically designed to acquire a predetermined amount of blood, which can be 10, 20, or 30 μL, and distinguishes itself from conventional dried blood spots (DBS) by enabling direct application to the fingertip. This method not only reduces the likelihood of imprecision, but also enhances feasibility, especially when samples are collected without supervision [5]. This technique is particularly useful for practical applications because of its minimal invasiveness and is well suited for frequent measurements in both scientific studies and clinical settings. For example, it can be employed in longitudinal studies or in studies with large sample sizes.

Therefore, this study aimed to develop and validate an LC-HRMS/MS-based assay optimized for the simultaneous quantification of eight relevant steroids and thyroid hormones in VAMS^®^ samples. This assay includes the hormones Δ4-androstenedione (A4), cortisol (F), 17β-estradiol (E2), dehydroepiandrosterone sulfate (DHEA-S), progesterone (P4), testosterone (T), triiodothyronine (T3), and thyroxine (T4). Hormonal parameters were selected based on the existing literature and consensus statements regarding endocrine changes resulting from chronic LEA. Studies in this area typically investigate controlled LEA conditions over short periods of time, such as several days, in controlled laboratory settings [1,2,6,7].

Additionally, this study aimed to determine whether VAMS^®^ samples and serum concentrations obtained using classical venipuncture provide comparable results.

## 2. Methods and Materials

### 2.1. Chemicals and Reagents

Mitra^®^ devices with VAMS^®^ technology (30 μL) were acquired from Neoteryx (Torrance, CA, USA).

Microlet^®^ lancets were obtained from Bayer Health Care (Leverkusen, Germany). Methanol (MeOH), acetone, *tert*-butyl methyl ether (TBME), NaHCO_3_, phosphate-buffered saline (PBS) (pH 7.4), and formic acid were purchased from Merck (Darmstadt, Germany). 1,2-dimethyl-1H-imidazole-5-sulphonyl chloride (DMIS) was purchased from Toronto Research Chemicals (Toronto, ON, Canada). All the chemicals and reagents used in this study were of analytical grade and were not further purified. Distilled and deionized water used in this study was generated using a Barnstead^TM^ GenPure^TM^ xCAD Plus system (Thermo Scientific^TM^, Waltham, MA, USA).

Steroid reference material and deuterium-labeled internal standards (ISTDs) A4, cortisol, cortisol-d4, DHEA-S, P4, progesterone-d9, T, testosterone-d3, E2 and 17β-estradiol-d4 were purchased from Toronto Research Chemicals (Toronto, ON, Canada).

Thyroid reference materials, including 3,3′,5-triiodo-L-thyronine (T3) (100 μg/mL) and L-thyroxine (T4) (100 μg/mL) and their internal standards 3,3′,5-triiodo-L-thyronine-^13^C_6_ (T3-^13^C_6_) (100 μg/mL) and L-thyroxine-^13^C_6_ (T4-^13^C_6_) (100 μg/mL), were obtained from Cerilliant^®^ (Round Rock, TX, USA).

### 2.2. Stock and Working Solutions

Stock solutions of A4, F, DHEA-S, E2, P4, T3, T4, and T (each 100 μg/mL) were prepared in MeOH and stored at −20 °C. Working solutions were prepared by diluting stock solutions to 10, 1, and 0.1 μg/mL, respectively.

Internal standard stock solution (ISTD) of cortisol-d4, progesterone-d9, T3-^13^C_6_, T4-^13^C_6_, and testosterone-d3 was prepared in MeOH at 0.01 μg/mL. As an internal standard for E2, a 17β-estradiol-d4 internal stock solution (ISTD) at 0.001 μg/mL was prepared freshly in deionized water.

### 2.3. VAMS^®^ Sample Preparation

For all method development experiments and validation steps, it was necessary to use steroid- and thyroid hormone-free samples, referred to as the blank samples. As commercially available hormone-free serum or VAMS^®^ samples were not available, whole-blood samples were collected from healthy volunteers and processed to create a surrogate matrix [8].

To prepare VAMS^®^ samples, red blood cells were separated from the serum by centrifugation for ten minutes at 2500× *g* using a Pico 17 Microcentrifuge (Thermo Fisher Scientific, Bremen, Germany). Following the removal of serum, red blood cells were washed with PBS (1:1, *v*/*v*) and centrifuged again for ten minutes at 2500× *g*. The upper phase was then carefully removed. This process was repeated six times to ensure the complete removal of all relevant hormones.

The cleaned red blood cells were then mixed with human serum albumin (Humanalbumin 5%, Biotest, Dreieich, Germany) (40:60 *v/v*) to a standardized hematocrit level (Hct) of 40%. The albumin was previously tested for the absence of all target analytes. Blank VAMS^®^ samples (30 μL) were prepared by gently mixing the hormone-free whole blood.

Calibrator VAMS^®^ samples were prepared by mixing the cleaned red blood cells with seven commercially available serum calibrators, including one blank calibrator, for A4, DHEA-S, E2, P4, and T. After following the manufacturer’s instructions to reconstitute the lyophilized serum controls from Chromsystems (Munich, Germany) in ultrapure water, seven calibrators were prepared by combining cleaned red blood cells with standardized serum to reach a Hct of 40%.

For the analytes F, T3, and T4, serum calibrators were spiked accordingly using the stock solutions. In addition to the calibrators, quality controls (QC II) in a medium concentration range were prepared in the same way as described above.

The tabulated data for the final VAMS^®^ concentrations of all analytes in the seven calibrators and QC II, which encompass a total volume of 30 µL including the serum compartment and red blood cells (RBCs), can be found in Table S1.

In order to compare the total capillary blood VAMS^®^ concentrations with the serum concentrations, the RBC fraction-corrected VAMS^®^ concentrations were determined. These corrected concentrations adjust for a 60% serum fraction of all VAMS^®^ samples, which corresponds to 18 µL of the 30 µL VAMS^®^ volume, when standardized to a Hct of 40%.

It was necessary to further take into account the proportion of the analyte in each compartment of whole blood, serum, and RBCs to convert total VAMS^®^ concentrations to RBC fraction-corrected VAMS^®^ concentrations. Taves et al. [9] established a plasma-to-RBC ratio for the analytes testosterone (4:1) and corticosterone (5:1); these were applied for A4, DHEA-S, T, and F, respectively.

The plasma-to-RBC ratio for T3 is reported as 3.2:1 [10] and the concentration of T4 in RBCs is 0.30–0.78 ng/mL, of which the mean value of 0.54 ng/mL was applied [11], accounting for less than 0.1% of the expected T4 concentration in the serum.

Using the aforementioned analyte-specific ratios, the present study calculated serum concentrations (*c_serum_*) from the total VAMS^®^ concentrations (*c_VAMS_*) using the following formula:

$$c_{VAMS}=0.6\times \;c_{serum}+0.4\times c_{RBC}$$

This leads to the equation for calculating *c_serum_* from VAMS^®^:

$$c_{serum}=\frac{c_{VAMS}-0.4\times c_{RBC}}{0.6}\;$$

using the known analyte-specific plasma-to-RBC ratio to obtain the RBC concentration (*c_RBC_*).

RBC fraction-corrected VAMS^®^ concentrations of all analytes are displayed in Table S2 (Supplementary Materials).

#### 2.3.1. VAMS^®^ Sample Extraction

After following the manufacturer’s instructions to prepare VAMS^®^ whole-blood samples, they were allowed to dry for a minimum of three hours at room temperature. Subsequently, they were individually wrapped in Ziploc bags with silica gel desiccants and stored at −80 °C until analysis. For analysis, the sample tips were removed from the plastic mount and placed in a 2.0 mL Eppendorf tube (Hamburg, Germany).

For steroid and thyroid hormone analyses, 300 μL of ISTD working solution in methanol was added to the sample tip. Target analytes were extracted using an ultrasonic bath for 30 min. After transferring the supernatant to a fresh 1.5 mL Eppendorf tube, the solvent was evaporated using a vacuum centrifuge at a temperature of 45 °C. The resulting dry residue was reconstituted in 100 μL of a mixture of methanol and water (50:50, *v*/*v*) and centrifuged for five minutes at 17,000× *g*. After transferring the supernatant into a glass injection vial, 30 μL was injected into the LC-HRMS/MS system.

For E2, extraction, 300 μL of ISTD working solution in water was added to a second sample tip, followed by 30 min of extraction in an ultrasonic bath. The supernatant was transferred into a fresh 2.0 mL Eppendorf tube together with 1.0 mL TBME for liquid–liquid extraction. After vortexing, the tubes were centrifuged for five minutes at 17,000× *g*. Subsequently, the upper phase was transferred into a fresh 1.5 mL Eppendorf tube and evaporated to dryness using a vacuum centrifuge at a temperature of 45 °C.

Afterwards, derivatization was performed by reconstituting the dry residue with 20 μL of DMIS (1 mg/mL in acetone) and 30 μL of NaHCO_3_ (50 mM in water) in a ThermoMixer (Eppendorf AG, Hamburg, Germany) at 65 °C and 600 rpm for 15 min. Finally, 30 μL was injected into the LC-HRMS/MS system.

#### 2.3.2. Venous Blood Sample Collection and Preparation

Venous blood was collected in K3-EDTA tubes (S-Monovette^®^, EDTA K3E/2.6 mL, Sarstedt, Inc., Nümbrecht, Germany) by a trained phlebotomist at the laboratory. Within 30 min of collection, serum was separated from red blood cells by centrifugation at 2500× *g* for 10 min. Serum was transferred into a fresh 0.5 mL Eppendorf tube and stored frozen at −20 °C until analysis.

For analysis, 100 μL of serum was diluted with 200 μL of acetic acid (1%), and 5 μL of the internal standard solution (20 ng/mL in MeOH) was added. After vortexing, the samples were equilibrated for 5 min at room temperature. Afterwards, 400 μL of acetonitrile was added to the sample and vortexed before the sample was shaken well in a ThermoMixer (Eppendorf AG, Hamburg, Germany) at room temperature at 600 rpm for 10 min. Finally, the sample was centrifuged at 17,000× *g* for 7 min and 150 μL was transferred into a glass injection vial. A volume of 10 μL was injected into the LC-HRMS/MS system.

### 2.4. Instrumentation and Analytical Conditions

All hormones were measured by LC-HRMS/MS. Analysis was performed using a Vanquish LC system coupled with an Orbitrap Exploris^TM^ 480 mass spectrometer (Thermo Fisher Scientific, Bremen, Germany). The LC system was equipped with an Agilent Poroshell 120 EC-C18 column (3.0 × 50 mm, 2.7 μm, Santa Clara, CA, USA) and the analytes were separated chromatographically with 0.1% formic acid as solvent A and methanol and 0.1% formic acid as solvent B. The flow rate was set to 400 μL/min. The gradient started at 10% B, was held for 1 min, and increased to 100% within 9 min. After a 3 min hold, it decreased to 10% B, followed by a re-equilibration phase at the starting conditions (3 min). The total run time was 16 min. The MS was operated in the polarity switching ionization mode (ESI+/ESI−) with a heated electrospray ion source (H-ESI), with an ionization voltage of 3000 V for ESI+ and −2600 V for ESI−. The vaporizer temperature was 300 °C and the ion-transfer capillary was heated to 320 °C.

Basically, three mass spectrometry (MS) settings were employed: a full scan experiment covering a range of *m*/*z* 100–800 at a resolution of 60,000 full width at half maximum (FWHM) and two targeted MS2 experiments, one in positive ionization mode (including all precursors on positive mode) and one in negative ionization mode. The precursor ion isolation window was adjusted to 1.5 Da, and the product ion mass spectra were acquired at a resolving power of 15,000 FWHM with individually optimized collision energies for each analyte. Table 1 summarizes the main LC-MS characteristics of the target analytes and product ions used as quantifiers and qualifier ions. The instrument was calibrated according to the manufacturer’s instructions.

### 2.5. Quantification/Statistical Evaluation of Data

Standard curves were constructed by plotting the peak area ratios of the respective hormones and their internal standards (ISTDs). To achieve this, seven calibrator samples, including one blank sample, were prepared, as previously described.

### 2.6. Method Validation

Method validation was performed based on U.S. Food and Drug Administration (FDA) guidelines for bioanalytical method validation [12]. These include parameter selectivity, linearity, imprecision, accuracy, limit of quantification (LOQ), recovery, matrix effects, carryover, robustness, and stability.

#### 2.6.1. Selectivity

Selectivity was assessed by the analysis of ten VAMS^®^ samples from healthy volunteers using the described protocol to identify the presence of possible interfering signals from the matrix or other sample components at the expected retention times.

#### 2.6.2. Linearity and Calibration Curve

Standard calibration curves were prepared using the Calibrator VAMS^®^ samples at seven different concentration levels, one of which served as a blank calibrator, as described in Section 2.3. To ensure that the relevant concentration range for each analyte was covered, the linearity of each analyte was individually assessed. The range was considered linear if the determination coefficient (R^2^) exceeded 0.99, and no systematic pattern was evident in the residuals.

#### 2.6.3. Imprecision

Intra- and interday imprecisions were assessed using VAMS^®^ samples with known concentrations in a medium working range (QC II). In three independent analytical runs, six VAMS^®^ replicates at one concentration level were prepared and analyzed according to the described method. The intraday and interday imprecisions were determined using the peak area ratios of one or three runs and their respective bandwidths. A 15% coefficient of variation (CV) was deemed acceptable for both intraday and interday imprecision [12].

#### 2.6.4. Accuracy

Accuracy was assessed by analyzing six VAMS^®^ replicates of a QC at a medium concentration level (QC II) of all analytes together with a calibration curve. The level of accuracy was determined by comparing the experimental and theoretical concentrations, as specified by the FDA, and a deviation of less than 15% from the target concentration was considered acceptable [12].

#### 2.6.5. Limit of Quantification (LOQ)

To estimate the LOQ, six VAMS^®^ replicates were prepared at five concentration levels within the range of the lowest concentration observed in the calibration curve. The LOQ was defined as the lowest concentration at which the analyte could be accurately quantified using a signal-to-noise ratio (SNR) of at least 10:1. According to the FDA, a coefficient of variation (CV) of 20% is an acceptable threshold for LOQ [12].

#### 2.6.6. Recovery

Six VAMS^®^ samples were prepared, each containing standard amounts of all analytes at concentrations of 20 ng/mL and 200 ng/mL for DHEA-S. Additionally, a separate set of six blank VAMS^®^ samples was prepared and fortified with 20 ng/mL of all analytes and 200 ng/mL of DHEA-S, immediately before being injected into the LC-HRMS/MS system. The relative recoveries of each analyte were calculated as a percentage of the ratio of the peak areas of the two sample sets.

#### 2.6.7. Matrix Effects

To evaluate the influence of ion suppression or enhancement resulting from the co-elution of matrix components, the matrix effect (ME) was determined. This was achieved by comparing the ratio of the mean peak area of the four spiked matrix extracts (A) to the four spiked solvent extracts (MeOH-H_2_O, 50:50, *v/v*) (B). The results are expressed as a percentage of A/B.

#### 2.6.8. Carryover

Carryover effects were evaluated by preparing two blank VAMS^®^ samples and a fortified VAMS^®^ sample in the upper working range of all analytes (50 ng/mL for all analytes except DHEA-S (200 ng/mL)) using the method described above. The fortified samples were injected into the LC-HRMS/MS system between blank samples. Carryover was evaluated through visual inspection of the chromatograms.

#### 2.6.9. Robustness

To assess the robustness of the method, the effects of the Hct (30–70%), prolonged extraction time (45 and 60 min), prolonged derivatization duration (45 and 60 min), and different derivatization temperatures (30 and 45 °C) were examined.

#### 2.6.10. Stability

To assess the stability of VAMS^®^ sample storage, 28 VAMS^®^ samples were prepared, each spiked with 20 or 200 ng/mL of DHEA-S. The samples were stored for varying periods of time, including 0, 1, 3, 5, 7, 14, and 28 days, at both room temperature (20 °C) and frozen (−18 °C) conditions until analysis. For each storage condition, VAMS^®^ samples were prepared in quadruplicate: two samples for each assay, steroids, thyroid hormones, and E2. To evaluate the degradation of the analytes, a concentration–time curve was generated for each sample within 28 days to determine the stability of all analytes. Stability was evaluated based on the FDA acceptance criterion of ±15% [12].

### 2.7. Comparison of Serum and VAMS^®^ Samples

To compare serum and VAMS^®^ samples, capillary blood samples were collected from healthy volunteers (*n* = 11, females *n* = 10, males *n* = 1) using Mitra^®^ tips, along with a corresponding serum sample. These paired samples were analyzed in duplicate during the same analytical run. Subsequently, the concentrations of VAMS^®^ samples were converted, as described in Section 2.3, to serum equivalents for comparison. Agreement between the two methods was assessed using Bland–Altman plots, while differences were evaluated using paired *t*-tests. Normal distribution of the data prior to analysis was assessed using the Shapiro–Wilk test.

### 2.8. Proof of Concept

To test the validated method for practical application, authentic VAMS^®^ samples from 50 athletes, of which 32 were female and 18 were male, were prepared according to the manufacturer’s instructions and analyzed using the developed method.

All volunteers signed a consent form and were instructed by a trained researcher via a video tutorial to prepare one VAMS^®^ sample, the collection of which was also supervised by a trained researcher. Female volunteers were instructed to prepare an additional VAMS^®^ sample for E2 analysis. The samples were collected in the morning at 7:30 h ± 15 min. Menstruating women were instructed to time the appointment in the late luteal phase, seven days after ovulation. Ovulation was determined individually by measuring urine luteinizing hormone (LH) concentration using semiquantitative ovulation tests. Capillary blood was collected via finger prick using Microlet^®^ lancets (Bayer Health Care, Leverkusen, Germany).

All procedures involving human participants were conducted in accordance with the ethical standards established by the institutional research committee and the 1964 Helsinki Declaration and its subsequent amendments. The local ethics committee of the German Sport University Cologne (#131/2023) approved the study, and written consent was obtained from all participating volunteers.

## 3. Results and Discussion

### 3.1. Method Development and Validation

**Figure 1:**
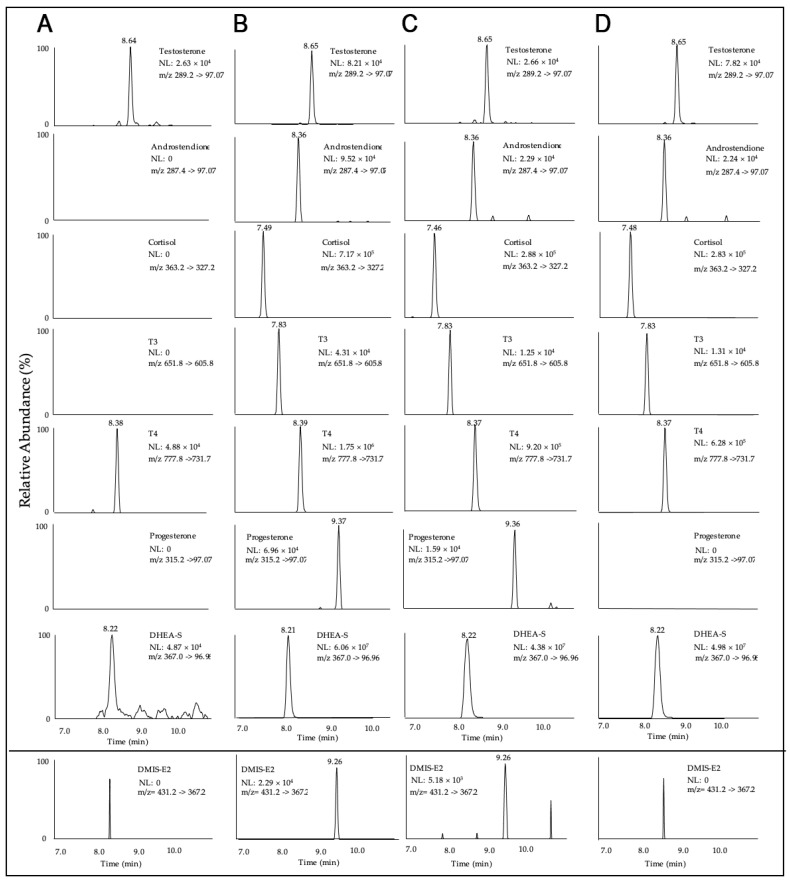
Extracted ion chromatograms of a blank (**A**), a calibrator level in the upper working range (**B**), an authentic female (**C**), and an authentic male (**D**) VAMS^®^ sample of all analytes.

To the best of our knowledge, the current assay represents the first method to measure eight steroids and thyroid hormones, including E2, simultaneously from two 30 μL Mitra^®^ devices with VAMS^®^ technology. The manual sample preparation was optimized with respect to the extraction agent used, extraction volume, and duration. Chromatograms of all analytes of a blank (A), a calibrator level in the upper working range (B), and an authentic female (C) as well as an authentic male (D) VAMS^®^ sample are shown in Figure 1.

A summary of all the validation results is presented in Table 2. In terms of selectivity, the ten samples displayed no interfering signals at the expected retention times of the analytes, owing to sufficient chromatographic separation. Calibration curves were found to be linear over the ranges of the seven individual calibrator concentrations, with R^2^ values greater than 0.99 for all analytes. Additionally, the intra- and interday imprecision results, as well as the accuracy results, met the FDA criteria, as the CV was below 15% for all analytes.

The method’s limit of quantitation (LOQ) was found to be suitable for assessing the levels of steroids and thyroid hormones in both male and female athletes. The LOQ of the steroids was lower than or in agreement with previously published LC-MS/MS methods using Mitra^®^ devices [13,14,15].

The recoveries for steroids were better than 63%. Although the recovery for both thyroid hormones was below 40%, it was considered acceptable, given the obtained LOQ for T3 and T4. Furthermore, all preparation steps were controlled by stable isotope-labeled internal standards to compensate for potential losses. It is common for extractions to be compromised when collecting blood from an absorptive material such as a polymeric tip or filter paper. This can occur because of interactions between the components of the sampling material and blood proteins, as well as coagulation of whole blood in a dried condition. However, in this assay, the most effective solvents and conditions were selected for extraction of the target analytes.

Matrix effects, ranging from 85 to 92%, indicated potentially low ion-suppression effects in the retention time window of the target analytes. The incorporation of a deuterium-labeled internal standard should effectively eliminate any potential effects that may arise from sample preparation, subsequent chromatographic separation, and mass spectrometric detection processes. The carryover of the method was negligible (<0.01%) for all analytes.

The long-term storage stability of the Mitra^®^ devices was evaluated over a period of 28 days at two distinct temperatures (20 °C vs. −18 °C). After 28 days of storage at −18 °C, no significant degradation (<11%) was detected for any of the analytes. At room temperature, the concentrations of the analytes after 28 days of storage were at least 85% of their initial values, except for F, which showed a degradation of less than 15% after 14 days of storage.

These findings support the suitability of Mitra^®^ devices with VAMS^®^ technology for autonomous sampling outside the laboratory; specifically, when samples are stored frozen after collection or transported to the laboratory via mail or any other means to be stored frozen within 14 days at room temperature.

In terms of robustness, no variations in extraction time, derivatization duration, or derivatization temperature had any impact on the results. However, differences in the concentrations of all the target analytes were observed within the Hct range of 30–70%. Because a Hct of 40% was used as the standardized value for preparing all calibrators and QC II in the present study, the deviations in concentrations over the observed Hct range are presented as the difference in concentration at the normalized Hct level of 40%, with an acceptable cutoff level of ±20%. As shown in Table S3 in the Supplementary Materials, T, P4, and A4 met the criteria in the Hct range of 30–50%, whereas DHEA-S, T3, T4, and F fulfilled the criteria within the Hct range of 35–50%.

The results demonstrate that the current method is reliable when calibrators are prepared with a standardized Hct of 40% across the Hct range of healthy adults, which includes values ranging from 40% to 54% for males and 36% to 48% for females [16]. Nonetheless, during or after physical activity, changes in plasma volumes can result in an increase in the Hct [17]. A study by Belvirani et al. [18] revealed an increase of 11% when comparing pre-exercise Hct levels to post-exercise levels (47.4 vs. 52.7%). The Hct returned to resting levels within three hours of exercise.

Based on the results described above, the method fulfilled all criteria for the quantification of all analytes over the investigated concentration range.

To the best of our knowledge, the present method is the first to quantify T3 and T4 from 30 μL Mitra^®^ devices with VAMS^®^ technology using LC-HRMS/MS. Existing methods for quantifying thyroid hormones from microsampled specimens utilize DBS, mainly in infants [19,20,21].

Iwayama et al. [21] measured T3, T4 and reverse T3 in DBS (spots of 8 mm diameter), equaling 12.5 µL of serum, using LC-MS/MS. For extraction, 2% NH_4_OH in ethanol was used, enabling a sensitivity that allowed measurement of T3 and T4 concentrations in infants (0.57–1.19 ng/mL for T3 and 79–157 ng/mL for T4).

The present assay reached an LOQ of 0.1 ng/mL for T3 and 0.5 ng/mL for T4, respectively, and was capable of reliably measuring T3 and T4 concentrations in all healthy athletes analyzed (Table 2). In the context of the RED-S, where thyroid hormones can be decreased, it is essential that an assay reliably detects concentrations below the reference ranges for healthy adults. The present validation results confirmed sufficient sensitivity to detect decreasing T3 and T4 concentrations. In particular, low circulating concentrations of T3 in the subnanogram per milliliter range are critical. Studies analyzing T3 concentrations in athletes with an increased risk for RED-S showed that the lowest T3 concentrations were ranging between 0.8 ng/mL [22] and 0.9 ng/mL [23] in athletes with low energy availability.

To explore changes in the hypothalamic–pituitary-thyroid axis related to RED-S, it might be beneficial to consider incorporating additional markers of thyroid function such as thyroid-stimulating hormone (TSH), free T3 (fT3), or free T4 (fT4).

The primary reason for selecting T3 and T4 in the present assay was the magnitude of the existing evidence from studies that examined altered thyroid function in the context of RED-S. To date, many studies have shown strong evidence for decreased T3 concentrations in athletes with low EA in the context of RED-S [1,6,22,23,24,25], which is also reflected in the actual consensus statement by the IOC on RED-S [2]. Additionally, different studies have concluded that T3 is a useful marker of low EA compared to other thyroid markers [1,26].

Another important aspect of microsampling is the low sample volume. Because the sample volume is limited to 30 μL in the present assay, it is challenging to reach an adequate sensitivity to detect other markers for thyroid function, such as TSH, fT3, and fT4, ranging below those of T3 and T4, in the low picogram per milliliter range. Additionally, it is noted that, to date, evidence for the diagnostic utility of fT3, fT4, and TSH in the context of LEA is still limited [1,25,25,27,28,29].

Regarding the quantification of E2 from microsampled specimens, published methods have so far failed to reach the sensitivity of measuring circulating estrogen levels in real samples from healthy adults [30,31]. E2 reference ranges in premenopausal women range between 8.4 and 474 pg/mL in the follicular phase, 74.9 and 780.1 pg/mL at LH peak, and 25.8 and 528.7 pg/mL in the luteal phase. In postmenopausal women and men, E2 levels range between 1.1 and 10.4 pg/mL, and between 3.3 and 37.0 pg/mL, respectively [32]. With an LOQ of 0.4 ng/mL in this assay, it also failed to reliably reach the sensitivity to measure low E2 concentrations in males, postmenopausal women, or premenopausal women in the early follicular phase. However, this method is suitable for menstrual phase classification, as well as for measuring increasing E2 levels throughout the menstrual cycle, such as the late follicular phase or luteal phase.

Nys et al. [30] described a method for E2 quantification without derivatization from 10 μL Mitra^®^ devices with VAMS^®^ technology which yielded an LOQ of 2.5 ng/mL. The present method exhibits a higher sensitivity owing to the utilization of DMIS for derivatization. This may also be attributed to the threefold increase in sample volume. The use of DMIS compensates for the weak ionization efficacy of E2, which functions as a specific derivatization reagent. The derivatization reaction is straightforward and analogous to the widely known dansyl chloride reaction, allowing the selective derivatization of the phenolic hydroxyl group of E2. By monitoring E2-specific confirmatory ion transitions across a broad concentration range using a DMIS, the confidence in the selectivity of the method can be enhanced during routine use, as demonstrated by Keski-Rahkonen et al. [33].

### 3.2. Comparison of VAMS^®^ and Serum Samples

When it comes to whole-blood microsampling, Hct bias is often considered an issue compared to serum. Therefore, this study aimed to compare two sample types: serum in venous blood collected through venipuncture and capillary blood from the fingertip collected via Mitra^®^ tips. Total VAMS^®^ concentrations were converted to serum equivalents as described previously.

A direct comparison of serum and RBC fraction-corrected VAMS^®^ samples from 11 volunteers is shown in Table 3.

**Figure 2:**
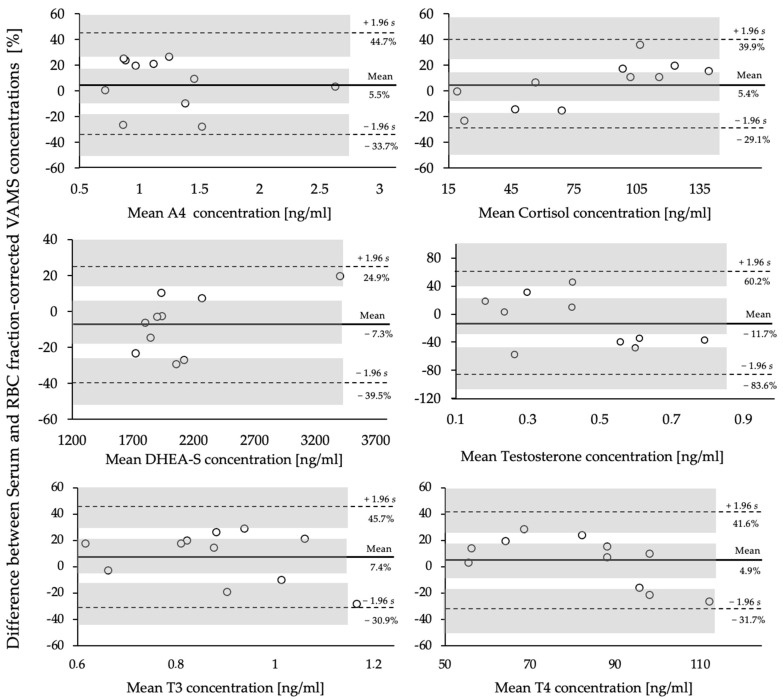
Bland–Altman Plots (*n* = 11) between serum concentrations and RBC fraction-corrected capillary VAMS^®^ concentrations, expressed as percentages of the differences, vs. the mean of both measurements for the analytes A4, cortisol (F), testosterone (T), DHEA-S, T3, and T4. Shaded areas represent 95% confidence interval limits for mean bias and limits of agreement (LoAs).

Figure 2 displays the Bland–Altman plots as percentages of the mean differences of capillary VAMS^®^ samples and venous serum samples of the six chosen analytes. Variations between both sampling methods showed positive and negative bias from the mean difference for all analytes, ranging between −92.15 and 2.00 ng/mL, even though they were not systematic. Results of the paired *t*-tests revealed no significant difference between both methods for all analytes (*p* > 0.05) (Table 3).

Although 95% of the data points for all analytes fell within the levels of agreement (LoA) (mean difference ± standard deviation), the clinical relevance of this variance, expressed as the maximum allowed differences between both methods, needs to be evaluated accordingly.

A potential reason for the discrepancies observed in the present pilot study may be the limited sample size (*n* = 11) of paired samples [34].

It is widely accepted that the greater the number of samples used for method evaluation, the narrower the resulting confidence intervals (CIs) for both the mean difference and the LoAs. Another potential reason for these discrepancies may be the structural difference between venous and capillary blood, as previously highlighted by Marshall et al. [9].

These results underline the existing difference between the matrices, serum, and VAMS^®^ samples and reinforce the need for proper and cautious interpretation.

To effectively employ Mitra^®^ devices with VAMS^®^ technology for RED-S assessment in practical applications, it is crucial to establish independent reference values for VAMS^®^ samples or to utilize standard serum reference values when a correction factor with a larger sample size of paired samples has been determined.

However, according to Ackermans et al. [35], addressing the issue of Hct is not crucial for hormones that predominantly exist in the plasma compartment to achieve a good correlation between plasma and DBS concentrations. Determining a conversion factor by measuring both plasma and DBS concentrations in a set of patient samples allows for the standardization of DBS concentrations to plasma concentrations, thereby simplifying the interpretation of obtained DBS concentrations. Hct can be measured directly or indirectly over a broad concentration range to establish a specific conversion factor. The use of this conversion factor in future analyses will ensure consistency in the results.

### 3.3. Proof of Concept

To demonstrate the efficacy of the presented approach, it is crucial to confirm the presence of substances not only in fortified samples, but also in authentic samples. To this end, a proof-of-concept experiment was conducted using authentic VAMS^®^ samples from healthy active adults (*n* = 50). The participant characteristics and mean RBC fraction-corrected hormone concentrations are summarized in Table 4.

Analytes A4, F, DHEA-S, T, T3, and T4 were quantified in all 50 samples, confirming the adequate sensitivity of the method.

These results are in agreement with those of previous studies that showed substantially higher T and DHEA-S concentrations in males [36,37]. A comparison between both sexes confirmed a significant difference in T levels (0.2 vs. 5.2 ng/mL, *p* < 0.001). The suitable sensitivity of the assay for T was corroborated by reliably quantifying T concentrations in all samples (male and female).

Both female sex hormones, E2 and P4, were detected exclusively in specimens obtained from female athletes. For female study volunteers, a measurement time point of seven days after ovulation was chosen, where both E2 and P4 were significantly increased compared to the follicular phase [38]. Although ovulation was determined individually using semiquantitative ovulation tests, E2 and P4 concentrations could not be quantified in all the female samples. The reasons for this may be individual irregularities in menstrual cycle length and, therefore, difficulties in applying ovulation tests accordingly.

The method developed can be employed for early assessments and prevention of RED-S, as well as in clinical contexts where the assessed hormones hold relevance.

Because of its minimal invasiveness and straightforward sample collection, it enables a higher frequency of measurements and, thus, less effort and stress for athletes. In the form of regular measurements of athletes, possibly at a higher risk of LEA, deviations in hormone levels over time can be detected and evaluated accordingly. During RED-S recovery, athletes’ progress may be monitored through regular hormone measurements by integrating microsampling into their daily lives. In this case, the effectiveness of a nutrition intervention can be monitored not only through anthropometric and performance parameters but also by normalizing hormone levels.

This study had some limitations that need to be acknowledged. One limitation was the lack of blank VAMS^®^ sample material for endogenous hormones. As illustrated in Figure 1A, the blank samples utilized for DHEA-S, T, and T4 were not entirely free of hormone residues. Although the whole-blood cleaning process described in Section 2.3 was intended to provide blank samples, it proved to be a challenge. When determining the concentration of endogenous hormone levels in the blank samples, the results were 45 ng/mL for DHEA-S, 0.001 ng/mL for T, and 0.15 ng/mL for T4. Because these concentrations account for less than 1% of the expected concentration ranges in authentic samples, they were considered acceptable. However, to quantify authentic samples, it is important to consider this bias by measuring blank samples in each analytical run and calculating the calibration curves accordingly. Another limitation is the sensitivity of E2 and P4, which is not sufficient to reliably quantify E2 and P4 levels in males generally or women in the follicular phase or menopause.

Finally, although a direct comparison of paired serum and capillary VAMS^®^ samples showed no systematic bias in the sample size (*n* = 11) used, it was not possible to establish a reliable correction factor for VAMS^®^ samples. For this, in addition to a larger sample size, assessments of individual Hct and the analytes’ specific distribution within the whole blood, considering different proportions in serum and RBCs, are required. As a result, the present concentrations cannot be directly compared to existing established analytes’ serum reference levels. This complicates the interpretation of the values obtained for VAMS^®^ samples. However, the intraindividual course of concentrations over a longer period can be reliably analyzed using the present method in a longitudinal study.

## 4. Conclusions

In conclusion, this study demonstrated the successful development and validation of an LC-HRMS/MS method to quantify eight steroid and thyroid hormones from two Mitra^®^ tips using Mitra^®^ tips with VAMS^®^ technology. The method is sensitive enough to reproducibly and accurately measure relevant hormones from spiked and authentic samples. The sensitivity showed suitability for quantification in healthy adults and allowed the measurement of decreasing concentrations in the context of RED-S.

Although serum and VAMS^®^ samples showed comparable results in the present assay, when used in sports practice or medicine, a direct comparison of paired serum and VAMS^®^ samples in a larger sample set and over a larger concentration range is warranted to adequately evaluate the obtained concentrations. Therefore, specific reference ranges for capillary blood or a reliable correction factor from serum to VAMS^®^ concentrations must be established for each analyte.

As regular hormone measurements in sports practice are sparse, microsampling techniques such as VAMS^®^ offer a practical alternative to venipuncture, as they facilitate the avoidance of extensive blood volume collection and the engagement of medical personnel, which can be both time-consuming and labor-intensive. In addition, this method offers new opportunities for research that require a higher frequency of measurement of decentralized studies in the field.

## Figures and Tables

**Table 1:** Analytical parameters and mass transitions of all analytes and internal standards.

Analytes	Precursor Ion [M+H]^+^ [*m*/*z*]	Precursor Ion [M-H]^−^ [*m*/*z*]	Quantifier Ion [*m*/*z*]	Qualifier Ion [*m*/*z*]	Retention Time (RT) [min]	Collision Energy (CE) [eV]
4-Androstenedione C_19_H_26_O_2_	287.2011		97.07	109.06	8.36	30
Cortisol (F) C_21_H_30_O_5_	363.2171		121.07	327.20	7.45	25
D4-Cortisol C_21_H_26_D_4_O_5_	366.2417		121.07		7.40	35
DHEA-S C_19_H_28_O_5_S		367.1578	96.96		8.22	35
Progesterone (P4) C_21_H_30_O_2_	315.2324		109.06	97.06	9.36	20
D9-Progesterone C_21_H_21_D_9_O_2_	324.2887		100.08		9.25	22
Testosterone (T) C_19_H_28_O_2_	289.2167		97.07	109.06	8.64	25
D3-Testosterone C_19_D_3_H_25_O_2_	292.2355		97.06		8.68	25
Triiodothyronine (T3) C_15_H_12_I_3_NO_4_	651.7978		605.79	507.87	7.83	20
C_13_T3 _13_C_6_C_9_H_12_I_3_NO_4_	657.7438		611.81		8.15	25
Thyroxine (T4) C_15_H_11_I_4_NO_4_	777.6866		731.69	633.76	8.37	25
C_13_T4 _13_C_6_C_9_H_11_I_4_NO_4_	783.7068		737.70		8.31	25
17β-Estradiol + DMIS C_23_H_30_N_2_O_4_S	431.1926		367.2		9.26	50
D4-Estradiol + DMIS C_23_D_4_H_26_N_2_O_4_S	435.2177		371.2		9.30	45

**Table 2:** Main validation results for all analytes.

Substance	Intraday Precision [%]	Interday Precision [%]	Accuracy [%]	Linearity [R^2^]	LOQ [ng/mL]	Recovery [%]	Matrix Effects [%]	Stability [Days]	
								(20 °C)	(−18 °C)
4-Androstenedione (A4)	5	7	87	0.99	0.5	81	91	>28	>28
Cortisol (F)	4	7	92	0.99	2.5	66	85	14	>28
DHEA-S	4	10	102	0.99	2.5	63	95	>28	>28
Progesterone (P4)	8	12	93	0.99	0.8	77	88	>28	>28
Testosterone (T)	2	3	96	0.99	0.02	84	92	>28	>28
Triiodothyronine (T3)	3	10	101	0.99	0.1	31	87	>28	>28
Thyroxine (T4)	5	9	119	0.99	0.5	27	89	>28	>28
17β-Estradiol + DMIS (E2)	3	4	108	0.99	0.04	87	90	>28	>28

**Table 3:** Comparison of serum and RBC fraction-corrected VAMS^®^ samples (*n* = 11).

Analyte	Serum [ng/mL]	VAMS^®^ [ng/mL]	Mean Bias [ng/mL]	95% Limits of Agreement (LoA)	Significance (*p*-Value)
A4	1.24 (±0.5)	1.19 (±0.6)	0.05	−0.40–0.50	0.457
F	81.54 (±46.6)	73.69 (±35.8)	−9.28	−19.97–38.54	0.130
DHEA-S	1932.07 (±675.4)	2050.00 (±496.0)	−92.15	−820.87–636.57	0.193
T	0.40 (±0.15)	0.48 (±0.27)	−0.10	−0.44–0.24	0.167
T3	0.92 (±0.17)	0.87 (±0.22)	0.06	−0.32–0.43	0.473
T4	84.76 (±15.5)	83.79 (±24.9)	2.00	−31.05–35.04	0.865

Serum and RBC fraction-corrected VAMS^®^ concentrations presented as mean values ± standard deviation (SD); 95% limits of agreement (LoA) calculated as the mean difference (bias) ± 1.96 times its SD.

**Table 4:** Subject characteristics and RBC fraction-corrected hormone concentrations of female and male participants in the proof of concept (*n* = 50). Data are presented as mean ± standard deviation.

	Male (*n* = 18)	Female (*n* = 32)	Significance (*p*-Value)
Age [years]	27.2 (±5.8)	24.0 (±5.0)	0.001
Height [cm]	182.8 (±7.0)	170.3 (±5.4)	0.001
Body Weight [kg]	80.8 (±13.6)	63.1 (±6.8)	0.001
BMI	24.1 (±3.5)	21.7 (±2.4)	0.009
A4 [ng/mL]	1.2 (±0.5)	1.7 (±0.7)	0.019
Cortisol (F) [ng/mL]	136.3 (±23.5)	136.5 (±39.5)	0.980
DHEA-S [ng/mL]	1826.7 (±843.8)	1535.8 (±728.4)	0.228
17β-Estradiol [ng/mL]	N/A	0.07 (±0.07)	N/A
Progesterone [ng/mL]	N/A	4.0 (±4.7)	N/A
Testosterone (T) [ng/mL]	5.2 (±1.4)	0.2 (±0.1)	0.001
T3 [ng/mL]	0.59 (±0.2)	0.56 (±0.1)	0.471
T4 [ng/mL]	37.3 (±12.6)	36.3 (±7.6)	0.728

## Data Availability

The raw data supporting the conclusions of this article will be made available by the authors upon reasonable request.

## Supplementary Materials

The following supporting information can be downloaded at: https://www.mdpi.com/article/10.3390/metabo14060328/s1, Table S1: Total VAMS^®^ concentrations of blank calibrators, Calibrator Levels 1–6 and Quality control (QC II) for all analytes; Table S2: RBC fraction-corrected VAMS^®^ concentrations of blank calibrators, Calibrator Levels 1–6 and Quality control (QC II) for all analytes; Table S3: Differences in analyte concentrations with varying hematocrit from 30% to 70%. Differences are presented as deviation in percent from a standardized hematocrit of 40%.
