# Supplementary material for: Development of an LC-HRMS/MS Method for Quantifying Steroids and Thyroid Hormones in Capillary Blood: A Potential Tool for Assessing Relative Energy Deficiency in Sport (RED-S)
Source: Metabolites. 2024 Jun 12;14(6):328. doi: 10.3390/metabo14060328 (PMC11205421; doi:10.3390/metabo14060328)
Supplement: Supplementary file 1 [file metabolites-14-00328-s001.zip › metabolites-3048790-supplementary.pdf]

Supplementary Materials

Table S1. Total VAMS® concentrations of blank calibrators, Calibrator Levels 1–6 and Quality control (QC II) for all analytes.

	Blank Calibrator	Calibrator Level 1	Calibrator Level 2	Calibrator Level 3	Calibrator Level 4	Calibrator Level 5	Calibrator Level 6	Quality control (QC II)
4-Androstendione [ng/ml]	< 0.0138	0.157	0.328	0.654	1.242	4.074	12.18	0.684
Cortisol [ng/ml]	< 1.5	12	30	60	120	180	240	60
DHEA-S [ng/ml]	< 14.64	61.2	344.4	654	1230	2254	3242	957
17β-Estradiol + DMIS [ng/ml]	< 0.015	0.029	0.077	0.194	0.386	1.164	3.858	0.248
Progesterone [ng/ml]	< 0.018	0.113	0.545	1.45	3.648	7.2	18.12	1.848
Testosterone [ng/ml]	< 0.003	0.040	0.188	0.75	2.25	4.476	8.82	0.906
T3 [ng/ml]	< 0.03	6	12	24	30	48	60	24
T4 [ng/ml]	< 0.3	0.15	0.3	0.45	0.6	1.2	3.0	0.45

Table S2. RBC fraction-corrected VAMS® concentrations of blank calibrators, Calibrator Levels 1–6 and Quality control (QC II) for all analytes.

	Blank Calibrator	Calibrator Level 1	Calibrator Level 2	Calibrator Level 3	Calibrator Level 4	Calibrator Level 5	Calibrator Level 6	Quality control (QC II)
4-Androstendione [ng/ml]	< 0.023	0.261	0.546	1.09	2.07	6.79	20.3	1.14
Cortisol [ng/ml]	< 2.5	20	50	100	200	300	400	100
DHEA-S [ng/ml]	< 24.4	102	574	1090	2050	3757	5403	1595
17β-Estradiol + DMIS [ng/ml]	< 0.025	0.048	0.129	0.324	0.644	1.94	6.43	0.414
Progesterone [ng/ml]	< 0.030	0.188	0.908	2.41	6.08	12.0	30.2	3.08
Testosterone [ng/ml]	< 0.005	0.067	0.313	1.25	3.75	7.46	14.7	1.51
T3 [ng/ml]	< 0.05	10	20	40	50	80	100	40
T4 [ng/ml]	< 0.5	0.25	0.5	0.75	1.0	2.0	5.0	0.75

Table S3. Differences in analyte concentrations with varying hematocrit from 30% to 70%. Differences are presented as deviation in percent from a standardized hematocrit of 40%.

Hematocrit	4-Androstendione	Cortisol	DHEA-S	17β-Estradiol + DMIS	Progesterone	Testosterone	T3	T4
30%	+20%	+32%	+31%	+20%	+19%	+ 20%	+38%	+37%
35%	+4%	+5%	+2%	+3%	-1%	+2%	+5%	+4%
40%								
45%	-2%	+2%	-1%	-2%	-7%	-4%	+1%	+2%
50%	-13%	-9%	-12%	-10%	-14%	-11%	-8%	-9%
55%	-30%	-31%	-36%	-26%	-38%	-28%	-37%	-31%
60%	-35%	-39%	-38%	-38%	-40%	-32%	-38%	-39%
65%	-43%	-44%	-49%	-45%	-47%	-40%	-47%	-44%
70%	-50%	-54%	-54%	-53%	-52%	-46%	-52%	-54%
